# Patientenberichtete Endpunkte – die Bedeutung der subjektiven Patientenperspektive für Forschung und klinische Versorgung

**DOI:** 10.1007/s00120-024-02405-4

**Published:** 2024-08-07

**Authors:** Andreas Dinkel, Matthias Jahnen

**Affiliations:** 1 Klinik und Poliklinik für Psychosomatische Medizin und Psychotherapie, Klinikum rechts der Isar, School of Medicine and Health, Technische Universität München, Langerstr. 3, 81675 München, Deutschland; 2 Klinik und Poliklinik für Urologie, Klinikum rechts der Isar, School of Medicine and Health, Technische Universität München, Ismaninger Str. 22, 81675 München, Deutschland

**Keywords:** Patientenberichtete Gesundheitsmerkmale, Routineversorgung, Patientenmonitoring, Prostatakarzinom, Patient-reported outcome measures, Patient-reported outcome measures, Delivery of health care, Patient monitoring, Prostate cancer, Health assessment

## Abstract

Symptome, Funktionsbeeinträchtigungen und die Lebensqualität können nur Patientinnen und Patienten adäquat beurteilen, die Einschätzung der Behandelnden stimmt diesbezüglich häufig nicht mit der von Betroffenen überein. Dem Rechnung tragend gewinnt die Berücksichtigung von patientenberichteten Endpunkten („patient-reported outcomes“, PROs) sowohl in der Forschung wie auch in der klinischen Routine an Bedeutung. In der praktischen Anwendung werden PROs nicht nur bei der Evaluation des Ergebnisses einer Behandlung berücksichtigt, sie können auch den Status vor einer therapeutischen Maßnahme beschreiben. Für die Erfassung der wichtigsten PROs liegen typischerweise mehrere reliable und valide Selbstbeurteilungsinstrumente vor, sog. „patient-reported outcome measures“ (PROMs). Im klinischen Alltag können PROs z. B. für die Diagnostik und Behandlungsplanung oder im Rahmen des Qualitätsmanagements genutzt werden. Das routinemäßige Patientenmonitoring mittels digitaler Medien (ePROMs) stellt gegenwärtig das vielversprechendste und am meisten beachtete Anwendungsgebiet in der klinischen Routine dar. Systematische Übersichtsarbeiten zeigen, dass die routinemäßige Erfassung von PROs u. a. mit einer Verbesserung der Arzt-Patient-Kommunikation, gesteigerter Patientenzufriedenheit, Symptombesserung, höherer Lebensqualität und einer höheren Überlebensdauer assoziiert ist. Dies gilt insbesondere dann, wenn die Ergebnisse des PRO-Monitorings an die Behandelnden zurückgemeldet werden. Trotz des inzwischen von vielen erkannten Nutzens von PROs und PROMs und der hohen Bereitschaft von Patienten, Angaben zu subjektiven Gesundheitsmerkmalen zu machen, gibt es noch zahlreiche Hürden bei deren Implementierung.

## Abstract (en)

Because only patients can adequately assess symptoms, disability, and quality of life, concordance between a patient’s and physician’s assessment is often low. Accordingly, patient-reported outcomes (PROs) are increasingly used in research and routine clinical care. In daily practice, PROs are not only applied to measure the patient’s perceived outcome of medical treatments, but also to assess their health status before intervention starts. Typically, several patient-reported outcome measures (PROMs), which are reliable and valid, are available for the assessment of the most important PROMs. In daily clinical practice, the integration of PROs can be useful for clinical assessment and treatment planning or for quality management. Currently, the most promising application is routine patient monitoring using digital PROMs (ePROMs). Systematic reviews have revealed that the routine use of PROMs in daily clinical care is associated with, among others, improved physician–patient communication, higher patient satisfaction, reduced symptom burden, higher quality of life, and improved survival. This effect is especially strong if health care professionals continuously receive the results of the PRO monitoring. Patients are usually inclined to disclose their health status, and the positive effects of routine patient monitoring are widely recognized. However, several barriers to using PROs and PROMs still exist.

Patientenzentrierung in der Medizin bedeutet, sich an dem subjektiven Befinden, den Bedürfnissen und den Werten der Patienten und Patientinnen zu orientieren. Über diese subjektiven Merkmale können die Patientinnen und Patienten selbst am besten Auskunft geben. Ein patientenberichteter Endpunkt („patient-reported outcome“, PRO) ist ein solches subjektives Merkmal. Dieses kann als Selbstbericht mittels Fragebogen erhoben werden. Dieser Selbstbeurteilungsbogen wird als „patient-reported outcome measure“ (PROM) bezeichnet. In den routinemäßigen Einsatz von PROMs im klinischen Alltag wird von einigen viel Hoffnung gelegt: „PROMs could help transform healthcare“ [8].

## Behandlerperspektive – Patientenperspektive

Der Erfolg einer medizinischen Behandlung, das Auftreten von Nebenwirkungen, der allgemeine Gesundheitszustand – dies wurde lange Zeit in letzter Instanz von Ärztinnen und Ärzten beurteilt. Die in der Gebrauchsinformation eines Medikaments aufgeführten möglichen Nebenwirkungen basierten früher nahezu ausschließlich auf der Beobachtung und Einschätzung von Ärzten und Ärztinnen. Die implizite Annahme hierbei ist, dass Behandelnde das subjektive Erleben von Patienten einschätzen und Symptome korrekt registrieren können [6]. Zahlreiche Studien widerlegen diese Annahme: Die Übereinstimmung der Symptomeinschätzung zwischen Behandelnden und Patienten ist niedrig bis höchstens moderat. Dies gilt sowohl für die Beurteilung unerwünschter Ereignisse in klinischen Studien [4, 12] als auch für die Einschätzung von körperlichen und psychischen Symptomen in der Routinebehandlung [6, 15, 20].

Behandelnde tendieren dazu, das Auftreten von Symptomen und deren Schweregrad zu unterschätzen

Behandelnde tendieren dazu, das Auftreten von Symptomen und deren Schweregrad zu unterschätzen [15, 25].

## Die formale Einführung von PRO

Es ist natürlich nicht korrekt zu sagen, dass vor dem Jahr 2009 keine PROs berücksichtigt wurden. Beispielsweise zeigte eine Metaanalyse aus dem Jahr 2008, in der die Ergebnisse von 39 klinischen Studien kondensiert wurden, dass PROs, und hier v. a. die gesundheitsbezogene Lebensqualität, einen Zusammenhang mit der Mortalität von Krebspatienten aufweisen [19]. Und Lohr und Zebrack [30] stellten 2009 fest, dass subjektive, patientenberichtete Gesundheitsmerkmale bereits seit mindestens 40 Jahren mittels Selbstbeurteilungsfragebögen erfasst werden.

Das Jahr 2009 ist allerdings insofern hervorzuheben, als zu diesem Zeitpunkt in den USA ein Bericht der Food and Drug Administration (FDA) erschien, der als sehr einflussreich zu werten ist [16]. Dieser Bericht enthält Empfehlungen für den Einsatz von PROs in klinischen Studien zur Arzneimittelwirkung. Demnach kann die Aussage, wonach ein Medikament einen Effekt auf ein Symptom zeigt, nur dadurch belegt werden, dass das entsprechende Symptom durch den betreffenden Patienten oder die Patientin selbst bewertet wird. Die FDA macht in ihrer Empfehlung deutlich, dass die Zulassung eines Medikaments auf Basis einer Symptombesserung, die alleine durch Patienten beurteilt wird, möglich ist. Der Fokus dieser Empfehlung ist recht eng gefasst, wie Basch [6] bemerkt. Beispielsweise fordere die FDA nicht, dass unerwünschte Ereignisse in klinischen Prüfungen durch die teilnehmenden Patienten berichtet werden müssen [6]. Allerdings enthält dieser Bericht auch eine Definition von PRO, die seitdem weithin zitiert wird, sowie u. a. auch Aussagen zu den Qualitätsanforderungen an Selbstbeurteilungsinstrumente zur Erfassung von PROs und Aussagen zum regelgerechten Vorgehen bei der Entwicklung von PROMs. Diese expliziten Aussagen und definierten Anforderungen bezüglich PRO und PROM hatten einen Einfluss, der über Arzneimittelstudien hinausging.

## Was genau sind PRO und PROM

Die Perspektive der Patienten und Patientinnen wurde in der Medizin regelhaft schon vor der Etablierung von PRO und PROM erfasst. In der Exploration und Anamnese kommt der Patient zu Wort und schildert Symptome und Funktionseinschränkungen. Diese werden durch den Behandelnden aber interpretiert: Der Arzt oder die Ärztin bewertet diese subjektiven Schilderungen vor dem Hintergrund der Ergebnisse der körperlichen Untersuchung, Laborparametern oder anderer Befunde.

Im Unterschied dazu geht es bei einem PRO ausschließlich um die Einschätzung des Patienten oder der Patientin, diese subjektive Einschätzung „ist“ das Erleben der Betroffenen und ist somit „wahr“.

Bei einem PRO geht es ausschließlich um die Einschätzung des Patienten oder der Patientin

Die FDA [16] definiert PRO als jedwede Angabe zum Gesundheitszustand eines Patienten oder einer Patientin, die direkt von der betroffenen Person stammt und nicht von den Behandelnden oder irgendjemand anderem interpretiert wird. Zu beachten ist, dass demnach nicht jede Selbstauskunft eines Patienten ein PRO darstellt, sondern nur solche, die den Gesundheitszustand betreffen. Psychologische Konstrukte wie beispielsweise Persönlichkeitsfaktoren oder soziale Unterstützung betreffen nicht die Einschätzung der Gesundheit und sind somit nicht als PRO anzusehen.

Der Begriff „Outcome“ (das „O“ in PRO) ist dahingehend ein wenig irreführend, als dass er nahelegt, es ginge nur um „End“-punkte, also um die subjektive Einschätzung von Symptomen nach einer medizinischen Behandlung, sozusagen um die subjektive Ergebnisbeurteilung der Behandlung. Historisch gesehen war dies der Zweck von PRO [13, 43]. Dieser Fokus auf Ergebnisbeurteilung mag auch der FDA-Handreichung geschuldet sein, die das Thema Nutzenbewertung von Arzneimitteln im Blick hatte und PRO aus einer Forschungsperspektive betrachtete, diese als Endpunkte klinischer Studien definierte [16].

Die Relevanz von PRO beschränkt sich jedoch nicht auf Studien, auch in der klinischen Routineversorgung werden PROs betrachtet und hier nicht ausschließlich als Evaluation am Ende der medizinischen Behandlung. Aus dieser klinischen Perspektive lässt sich ein PRO besser als ein „patientenberichtetes Gesundheitsmerkmal“ begreifen ([8, 28]; s. auch Charité Center for Patient-Centered Outcomes Research; https://cpcor.charite.de).

PROs werden mit Selbstbeurteilungsfragebögen (PROMs) erfasst

Typische patientenberichtete Gesundheitsmerkmale sind körperliche Symptome wie Schmerz oder Fatigue, psychische Probleme wie Angst, Depressivität und Distress sowie Funktionsbeeinträchtigungen wie Einschränkungen der körperlichen Funktionsfähigkeit. Das am häufigsten betrachtete PRO stellt sicherlich die gesundheitsbezogene Lebensqualität dar. Neben diesen generischen PROs, die krankheitsübergreifend relevant sind, lassen sich auch krankheitsspezifische PROs definieren.

Erfasst werden PROs mit Selbstbeurteilungsfragebögen (PROMs). Oft existiert für ein bestimmtes Gesundheitsmerkmal bereits eine Reihe an PROMs. Damit sie sinnvoll in Studien oder in der klinischen Routine eingesetzt werden können, müssen sie bestimmte psychometrische Qualitätsmerkmale erfüllen. Aber auch wenn PROMs als psychometrisch gut eingestuft werden können, heißt es nicht zwingend, dass ihr Einsatz unproblematisch ist. So zeigte die Studie von Wiering et al. [42] zu PROMs, an deren Entwicklung Patientenvertreter nicht beteiligt waren, dass betroffene Patienten einige Items der eingesetzten PROMs als weniger wichtig oder gar unwichtig erachteten. Dies stellt offenkundig ein Problem dar, da das Gesundheitsmerkmal somit nicht adäquat abgebildet wird. Die FDA [16] empfiehlt daher, PROMs unter Beteiligung von Patienten und Patientinnen zu entwickeln.

## Anwendungsfelder

Die PROs und PROMs können in unterschiedlicher Weise in Forschung und klinischer Praxis genutzt werden. Warsame und D’Souza [41] nennen als mögliche Anwendungsfelder Zulassungsstudien, klinische Studien, Studien zur vergleichenden Nutzenbewertung, Krebsregister und klinische Versorgung. Weitere Einsatzgebiete sind populationsbasierte Studien und Studien mit Personen, die nach und mit einer Krebserkrankung leben („cancer survivorship“; s. Infobox 1).

Im Rahmen von Zulassungsstudien können PROs als primäre, sekundäre oder explorative Endpunkte betrachtet werden [16]. Paravathaneni et al. [35] untersuchten die Studienprotokolle zu 40 von der FDA zugelassenen Medikamenten bei urogenitalen Tumoren, die meisten davon für das Prostatakarzinom [16]. Nur in 31 Trials (77,5 %) war geplant, PRO-Daten zu erheben. In keiner der Studien stellte ein PRO den primären Endpunkt dar. Für 27 Studien (87 %) lagen publizierte Ergebnisse zu PROs vor. Das am häufigsten untersuchte Konstrukt war die gesundheitsbezogene Lebensqualität. Die Autoren äußern sich insgesamt kritisch zum Trial-Design, der Handhabung der PROs als Endpunkte und der Qualität der Ergebnisdarstellung zu den PRO-Daten. Da beispielsweise über ein Drittel der Trials PROs als explorativen Endpunkt konzipierte, sollten die entsprechenden Ergebnisse und Signifikanzen nicht überbewertet werden.

Die PROs können in klinischen Studien aber auch für die Erhebung unerwünschter Ereignisse genutzt werden [6, 41]. Es liegt hierfür auch eine deutschsprachige PROM-Version des Standardinstruments Common Terminology Criteria for Adverse Events (CTCAE) vor [23], und seit kurzem sind tumorspezifische Versionen verfügbar, u. a. für das Prostatakarzinom [22].

Ferner bestätigte eine aktuelle systematische Übersichtsarbeit erneut, dass in klinischen Studien PROs mit der Überlebenszeit von Krebspatienten assoziiert sind. Vor allem die Variablen körperliche Funktionsfähigkeit und globale gesundheitsbezogene Lebensqualität erwiesen sich als bedeutsam. Daher könnten PROs in klinischen Studien auch als Prognosefaktoren oder Stratifizierungsmerkmale genutzt werden [33].

Bei Studien zur vergleichenden Nutzenbewertung verschiedener Behandlungsoptionen ist es essentiell, die Patientenperspektive zu berücksichtigen. Ohne diese hätten Patienten und Patientinnen, Forschende, Behandelnde, Kostenträger und Funktionäre keine ausreichende Grundlage, um Entscheidungen in Bezug auf Behandlungsoptionen zu treffen [41]. Ein Bespiel ist die ProtecT-Studie, die Patienten mit primär lokalisiertem Prostatakarzinom untersuchte. Diese wurden randomisiert den Behandlungsoptionen aktive Überwachung, radikale Prostatektomie oder Strahlentherapie zugewiesen. Die aktuelle Publikation zum15-Jahre-Follow-up zeigt keinen Unterschied in der prostatakrebsspezifischen Mortalität (dem primären Outcome) zwischen den drei Gruppen [24]. Somit lässt sich aufgrund dieses Ergebnisses keine der Behandlungsoptionen eindeutig bevorzugen.

Die Auswertung der PRO-Daten zum 12-Jahre-Follow-up der ProtecT-Studie erbrachte, dass einige Langzeitfolgen persistierten oder sich sogar verschlimmerten und dass diesbezüglich Unterschiede zwischen den Behandlungsgruppen bestanden. Beispielsweise trat Harnverlust nach radikaler Prostatektomie häufiger auf als nach Strahlentherapie oder aktiver Überwachung, während Stuhlinkontinenz nach Strahlentherapie doppelt so häufig berichtet wurde (12 %) wie in den beiden anderen Behandlungsarmen. Ferner stellen die Autoren fest, dass die sexuelle Funktionsfähigkeit in allen drei Gruppen niedrig war, jedoch ließ sich ein unterschiedliches Verlaufsmuster der Beeinträchtigung über die 12 Jahre beobachten [14]. Unterschiede in den PROs nach 10 Jahren fand z. B. auch eine Beobachtungsstudie, die die Folgen von radikaler Prostatektomie, Brachytherapie und externer Strahlentherapie verglich [17]. In der Konsequenz sprechen die Ergebnisse dieser Studien für die Notwendigkeit individualisierter Behandlungsentscheidungen, bei denen Patienten und Behandler die kurz-, mittel- und langfristigen Therapiefolgen abwägen müssen.

Die bereits erwähnte ProtecT-Studie wie auch andere Arbeiten haben gezeigt, dass Patienten mit Prostatakarzinom sehr hohe Überlebensraten haben. Dies heißt auch, dass immer mehr Personen als Langzeitüberlebende nach Prostatakrebs anzusehen sind. Wie Russell et al. [37] bemerken, mangelt es aber an Daten, v. a. Langzeitdaten, zur Lebensqualität dieser großen Gruppe an Prostatakrebsbetroffenen. Im Rahmen von PIONEER, einem europäischen Exzellenznetzwerk für „Big Data“ bei Prostatakrebs, wurde das Thema Survivorship als prioritär eingestuft, und es wurde ein Core Outcome Set definiert, das v. a. körperliche Symptome und die allgemeine Lebensqualität umfasst [37].

Die hier definierten Core Outcomes lassen das psychische Befinden unberücksichtigt, was ein wenig erstaunt. So konnten wir in einer Längsschnittstudie mit Langzeitüberlebenden nach Prostatektomie zeigen, dass Progredienzangst, typischerweise die stärkste emotionale Belastung nach der Diagnose einer Krebserkrankung, sich über die Jahre nur wenig verändert und auch noch über 15 Jahre nach Prostatektomie für einen Teil der Betroffenen ein großes Problem darstellt [32]. Im Vergleich zu dem PIONEER-Vorschlag ist das Core Set, das mittels eines Delphi-Prozesses verabschiedet wurde und an dessen Entwicklung auch von Krebs betroffene Personen mitwirkten, weiter gefasst. Neben Körperbeschwerden sind hier auch psychische und soziale Folgen der Krebserkrankung berücksichtigt ([36]; s. Infobox 2).

Nicht im Core Set enthalten und auch seltener in Studien untersucht sind PROs, die eine positive Adaptation an die Erkrankung und die Therapiefolgen betrachten. Beispielsweise konnten wir zeigen, dass es ca. 50 % der von Prostatakrebs betroffenen Männern gelingt, ein moderates bis hohes Maß an „benefit finding“ zu erleben, d. h. positive Konsequenzen aus den Folgen der Erkrankung zu ziehen [26].

### Klinische Versorgung

Im Rahmen der klinischen Versorgung können PROs auf vielfache Weise eingesetzt werden. Kowalski et al. [28] unterscheiden dabei zum einen zwischen dem Zweck der individuellen Patientenbetreuung und zum anderen dem der Qualitätsentwicklung. Andere Autoren erwähnen als Einsatzmöglichkeiten zudem psychosoziale Interventionen, v. a. eHealth-Interventionen zur Steigerung des Selbstmanagements, oder ein nutzenorientiertes Vergütungsmodell im Sinne von „Value Based Health Care“ [39].

Im Rahmen der individuellen Patientenbetreuung lassen sich die Anwendungsmöglichkeiten Diagnostik und Behandlungsplanung sowie Monitoring unterscheiden [28]. Ein Beispiel für Diagnostik und Behandlungsplanung ist das psychoonkologische Distress-Screening, das in Krebszentren, die von der Deutschen Krebsgesellschaft zertifiziert sind, routinemäßig durchgeführt werden muss (vgl. SOP Distress Screening; https://www.krebsgesellschaft.de/zertdokumente.html). Das Distress-Screening dient dazu, die psychische Belastung von Patienten und Patientinnen zu erfassen, die Indikation für eine psychologische Versorgung zu stellen und diese bei Bedarf in die Wege zu leiten [40]. Hierfür bedarf es u. a. definierte Verantwortlichkeiten, organisationale Routinen und klare Algorithmen der konsiliarischen Anforderung des psychoonkologischen Dienstes. Um diese Prozesse zu erleichtern, erscheint die Nutzung eines elektronisches Distress-Screenings (ePROM) unausweichlich [40].

Das Monitoring mittels PROMs hat seit der Studie von Basch et al. [7] deutlich an Interesse gewonnen. In dieser randomisierten Studie machten Patienten und Patientinnen der Interventionsgruppe sowohl zu den Konsultationsterminen als auch zwischen den Terminen Angaben zu ihren körperlichen Beschwerden (elektronische Erfassung). Wurden schwere Symptome oder eine Verschlechterung von Symptomen berichtet, erhielt die zuständige Pflegefachkraft eine Benachrichtigung per E‑Mail. Für den behandelnden Onkologen wurde bei jedem ambulanten Besuch ein Bericht generiert, der den Symptomverlauf für die betroffene Person darstellte. Das mittlere Überleben in der Gruppe mit Symptommonitoring war ca. 5 Monate länger verglichen mit der Kontrollgruppe, die die Routinebehandlung erhielt. Eine naheliegende Erklärung für diesen Effekt ist ein verbessertes Symptommanagement, da sich die benachrichtigte Pflegefachkraft aufgrund der erhaltenen Information mit den Betroffenen in Verbindung setzte und eine Optimierung der Behandlung besprach. Ein solches Monitoring ist offenkundig nicht nur in Bezug auf körperliche Symptome möglich, sondern auch im Hinblick auf psychische Beschwerden oder Medikationsadhärenz, was aktuell in verschiedenen Studien untersucht wird [28].

ePROMs stellen zzt. das vielversprechendste Anwendungsgebiet von PRO und PROM dar

Das routinemäßige Patientenmonitoring mittels ePROM stellt gegenwärtig das vielversprechendste und am meisten beachtete Anwendungsgebiet von PRO und PROM in der klinischen Routine dar [11]. Solch ein Monitoring sollte aber nicht nur auf Patienten und Patientinnen beschränkt sein, die sich in aktiver Therapie befinden. Eine aktuelle Leitlinie der European Society for Medical Oncology empfiehlt, ein Symptommonitoring mittels PROM auch in der palliativen Versorgung in Betracht zu ziehen, bei Patientinnen und Patienten, die sich am Ende ihres Lebens befinden. Zudem wird diese Empfehlung auch für die Nachsorge und für die Betreuung von Langzeitüberlebenden, die mit und nach einer Krebserkrankung leben, formuliert [11]. Als relevante patientenberichtete Gesundheitsmerkmale können dabei nach Singhal et al. [38] dieselben herangezogen werden, die bereits für die Survivorship-Forschung konsentiert wurden ([36], s. Infobox 2).

## Effekte von PRO und PROM in der klinischen Routine

Ein erster systematischer Review von Chen et al. [10] zu den Effekten einer routinemäßigen Erfassung von PROs erbrachte, dass dies deutlich mit einer Verbesserung der Arzt-Patient-Kommunikation und mit gesteigerter Patientenzufriedenheit assoziiert ist. Auch zeigten sich Hinweise in Richtung Symptombesserung. Diese Ergebnisse wurden in späteren systematischen Übersichtsarbeiten weitgehend bestätigt [18, 21, 29, 44] und um weitere Zusammenhänge ergänzt. So besteht Evidenz für eine verbesserte Lebensqualität [18, 21, 38], eine Abnahme ungeplanter Besuche in der Notaufnahme, seltenere Krankenhausaufnahmen [29] und eine höhere Überlebensdauer ([29, 31]; vgl. Infobox 3). Um möglichst deutlich positive Effekte zu erzielen, erscheint es notwendig, die Ergebnisse des routinemäßigen Patientenmonitorings kontinuierlich an die Behandelnden zurückzumelden [18, 21, 31].

## Implementierung

Patienten und Patientinnen sind – vielleicht entgegen der Erwartung mancher Behandler – bereit, auch umfangreiche Angaben zu Gesundheitsmerkmalen zu machen. Ein hoher Anteil bearbeitet PROMs nahezu vollständig [38]. Patientinnen und Patienten empfinden den Einsatz von PROMs als sinnvoll, und sie haben den Eindruck, dass sie selbst wie auch Ärztinnen und Ärzte besser auf die Konsultation vorbereitet sind [9].

Ein hoher Anteil von Patienten und Patientinnen bearbeitet PROMs nahezu vollständig

Atkinson et al. [5] fanden, dass Patienten eher wenig Belastung durch das Bearbeiten der PROMs empfanden und z. T. sogar bereit wären, noch weitere Items zu beantworten, sofern diese aus ihrer Sicht wichtig sind. Andererseits empfiehlt beispielsweise die FDA [16], Patienten durch den Einsatz von PROMs nicht übermäßig zu belasten. Diese Frage adressiert auch ein aktuelles Konsensusstatement einer internationalen Autorengruppe, in dem 19 Empfehlungen zum adäquaten Einsatz von PROMs unter dem Gesichtspunkt der zeitlichen und psychischen Anforderungen an Patienten gegeben werden [1].

Trotz des inzwischen von vielen erkannten Nutzens von PROs und PROMs und der hohen Bereitschaft von Patienten, Angaben zu Gesundheitsmerkmalen zu machen, gibt es noch zahlreiche Hürden bei deren Implementierung, sowohl in der Forschung als auch in der klinischen Routine. Diese Hürden können auf der Ebene der Patienten und Patientinnen, der Behandelnden und der Organisation bestehen [34]. Die aufzuwendende Zeit wie auch der Umgang mit elektronischen PRO-Systemen kann für Patienten wie auch für das betreuende Gesundheitspersonal ein Hemmnis darstellen [34]. Ebenso spielen mangelndes Wissen und skeptische Einstellungen bei den Behandelnden und Vertretern der Leitungsebene eine Rolle. Die Auffassung, dass PROMs primär eine Berichtspflicht darstellen und nicht ein Werkzeug der Qualitätsentwicklung steht einer erfolgreichen Implementierung im Wege [3].

Ein großes Problem stellen fehlende Ressourcen, eine ungenügende Integration der Erhebung von PROMs in bestehende klinische Arbeitsabläufe und eine inkompatible informationstechnische Infrastruktur dar [28, 34]. Da ein routinemäßiges Patientenmonitoring v. a. dann effektiv ist, wenn ein Feedback der Ergebnisse an die Behandelnden erfolgt, stellt sich zudem die Frage, wie die Ergebnisse sinnvoll rückgemeldet und visualisiert werden sollen. Werden Veränderungen in den PROM-Daten über die Zeit präsentiert, so stellt u. U. auch die korrekte Interpretation von Veränderungswerten als unbedeutend vs. klinisch bedeutsam eine Herausforderung dar [2, 27, 39].

### Infobox 1 Anwendungsfelder für PRO

- Klinische Studien,
- Studien zur vergleichenden Nutzenbewertung unter Routinebedingungen („comparative effectiveness research“),
- Krebsregister,
- populationsbasierte Studien zu „cancer survivorship“,
- klinische Versorgung.

### Infobox 2 Expertenkonsens zu einem PRO Core Set für die Cancer-Survivorship-Forschung [36]

- Depressivität,
- Angst,
- Schmerz,
- Fatigue,
- kognitive Beeinträchtigung,
- Progredienzangst,
- Rollenfunktion im Alltag,
- finanzielle Toxizität („financial toxicity“),
- Krankheitsbewältigungsverhalten,
- allgemeine Beeinträchtigung durch Nebenwirkungen,
- allgemeine Lebensqualität,
- allgemeiner Gesundheitsstatus.

### Infobox 3 Positive Effekte eines routinemäßigen PRO-Monitorings [10, 18, 21, 29, 31, 44]

- verbesserte Arzt-Patient-Kommunikation,
- höhere Patientenzufriedenheit,
- reduzierte Symptomlast,
- besserer Gesundheitszustand,
- höhere Lebensqualität,
- längeres Überleben,
- weniger ungeplante Besuche in der Notaufnahme,
- geringere Hospitalisierung,
- reduzierte Gesundheitskosten.

## Fazit für die Praxis

- Wesentliche Ziele medizinischer Behandlungen sind Symptomlinderung, Reduktion von Funktionsbeeinträchtigungen und Steigerung der Lebensqualität. Diese Aspekte können nur die betroffenen Patienten und Patientinnen selbst adäquat beurteilen.
- Die von den Patienten und Patientinnen berichteten Gesundheitsmerkmale („patient-reported outcomes“, PROs) sind subjektive Einschätzungen, die als „wahr“ zu werten sind und nicht durch Behandelnde interpretiert werden müssen.
- Für die Erfassung der wichtigsten PROs liegen in der Onkologie typischerweise mehrere reliable und valide Selbstbeurteilungsinstrumente vor („patient-reported outcome measures“, PROMs).
- Die Berücksichtigung von PROs in der Forschung und der klinischen Routine trägt zu einer besseren patientenzentrierten Medizin bei.
- Insbesondere das routinemäßige PRO-basierte Monitoring von Patienten und Patientinnen im klinischen Alltag hat das Potenzial, zu einer Verbesserung der Versorgung beizutragen.
